# What is in the drug packet?: access and use of non-prescribed poly-pharmaceutical packs (Yaa Chud) in the community in Thailand

**DOI:** 10.1186/s12889-019-7300-5

**Published:** 2019-07-22

**Authors:** Malee Sunpuwan, Sureeporn Punpuing, Wipaporn Jaruruengpaisan, John Kinsman, Heiman Wertheim

**Affiliations:** 1 Institute for Population and Social Research, Mahidol University, Salaya, Nakhon Pathom Thailand; 2 Department of Epidemiology and Global Health, Umeå University, Umeå, Sweden; 3 Department of Public Health Sciences, Global Health (IHCAR), Karolinska Institutet, Stockholm, Sweden; 4 Oxford University Clinical Research Unit, Hanoi, Vietnam; 5 The Nuffield Department of Medicine, University of Oxford, Oxford, UK; 6 Department of Clinical Microbiology and the Radboud Center for Infectious Diseases, Radboudumc, Nijmegen, Netherlands

**Keywords:** Yaa Chud, unknown medicine, Access, Use, Thailand

## Abstract

**Background:**

‘Yaa Chud’ is a non-prescribed poly-pharmaceutical pack containing several types of drugs, including antibiotics and steroids, which can be purchased over the counter in Thailand for self-medication. Although it is illegal, it is still available at some community outlets. This study aimed to understand access to and use of Yaa Chud at the community level in order to raise awareness on its usage and to provide policy recommendations to address the problem.

**Methods:**

This study employed qualitative methods, including in-depth interviews with 18 drug suppliers and 16 community members, and six focus group discussions. It included inventories from 17 drug suppliers. Data were collected in selected communities of the Kanchanaburi Demographic Surveillance System, located in the western region of Thailand.Thematic analysis was based upon the Health Services Utilization Model and conducted using the Open Code qualitative software program.

**Results:**

Overcrowding, long waiting times, and a perceived unwelcoming environment at public health-care service outlets were identified as factors that drive people into the private sector, where loose regulation of drug laws facilitates access and use of Yaa Chud. Migrants and older people were most likely to seek and use Yaa Chud, especially for mild illness. Availability, easy access through a user’s network, low cost, and perceived effectiveness were identified as factors that enable access and use of Yaa Chud.

**Conclusions:**

Though illegal in Thailand, Yaa Chud is likely to remain available for self-medication by community members, due to the persisting demand by the elderly and migrant workers. There is an urgent need to replace these mixed medications with better choices. Safer Yaa Chud may be a preferred, first-line health-care option, which could help reduce congestion in the formal health-care setting. At the same time, enforcement of regulatory compliance needs to be continued in order to stop the supply of unsafe Yaa Chud.

**Electronic supplementary material:**

The online version of this article (10.1186/s12889-019-7300-5) contains supplementary material, which is available to authorized users.

## Background

Self-medication is broadly defined as the use of a medicinal product in the absence of a consultation with a qualified clinician, and based on either a self-diagnosis or that of another unqualified individual [1]. Evidently the majority of people worldwide use self-medication to treat common health problems as their health-care practice and, as such, self-medication is recognized as the first line of health care [2, 3]. However, self-medication was recognized as an “unhealthy practice” in the 1960s [4], and ways have been sought to replace hazardous self-medication products with safe alternatives.

Currently, self-medication is widely used, in particular in many Low and Middle Income Countries (LMICs) [5] where informal health-care providers play an important role in health-care delivery, despite their lack of formal training or licensing [6]. Governments with interest in this issue often focus on unlicensed drug sellers who are then provided with information and training in order to improve access to safe medicines without a prescription [1]. If done properly, self-medication can benefit individuals and the health-care system. It also empowers the individual to take more responsibility for his or her own health and, as such, creates resilient communities [7]. However, several self-medication practices can be unsafe, and need to be regulated.

**Fig. 1:**
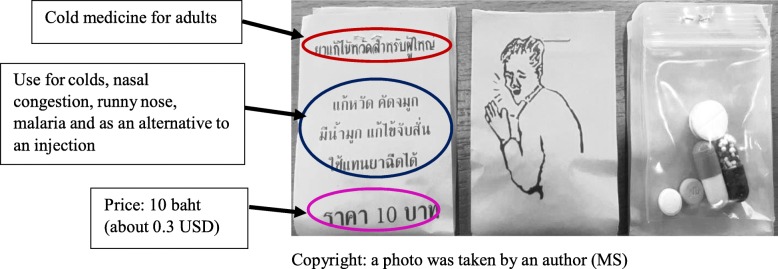
Characteristics of the non-prescribed poly-pharmaceutical packet: Yaa Chud

One form of self-medication in Thailand that is considered as an unhealthy practice is the use of “Yaa Chud” (ยาชุด), the focus of this study. Even though Thai people are under a government health-care scheme at low or no cost, some Thais still prefer self-medication and are even willing to pay out of their own pocket. National surveys in Thailand have found that self-medication rates are high, with 31–35% of respondents self-medicating within the past month [8, 9]. Moreover, Yaa Chud is one empirical option for self-medication [10, 11]. Yaa Chud is a non-prescribed poly-pharmaceutical pack, containing a set of medicines that are unidentifiable by the consumer and sold in neighbourhood pharmacy shops and grocery stores [10, 11]. Usually, Yaa Chud is a combination of several drugs, including capsules and tablets, which are packed together in small plastic bags. The label indicates the condition for which the combination of drugs can be used, but they do not include the drugs’ names (see Fig. 1). Yaa Chud are used in the community for pain, stomach upsets, colds, dysuria and poor appetite. Generally, one packet contains three to five pills, depending on the condition it is supposed to treat [12]. Laboratory tests in previous studies have found that there are several types of drugs in one packet. For instance, a study in one district in north-eastern Thailand found 51 different types of Yaa Chud packages for pain. Almost three-quarters of these Yaa Chud included steroids and non-steroidal anti-inflammatory drugs (NSAIDs) [13]. Another study in the central region found that slightly more than half of Yaa Chud for cold/flu symptoms contained at least one antibiotic, such as amoxicillin, ampicillin, metronidazole or chloramphenicol [14].

The prevalence of Yaa Chud has become a major public health concern in Thailand, since the packs often contain antibiotics, steroids and NSAIDs [11, 15], and they are widely available in community-based grocery stores [11, 16, 17]. Taking Yaa Chud may lead to erroneous dosing of antibiotics, thereby contributing to antimicrobial resistance [18]. Adverse effects of steroids are another concern [11, 19]. In Laos, Yaa Chud is also commonly sold for self-medication, however there is evidence that this can lead to serious adverse events, such as gastric ulcer [20].

The Thai government (Ministry of Public Health) is concerned about the prevalence of Yaa Chud, and has formulated a drug act as well as launching public campaigns in order to stop the use of Yaa Chud. The focus of the campaign is on the danger of Yaa Chud. An example of a campaign slogan is: “Yaa Chud Sud Antarai” (“ยาชุดสุดอันตราย =Yaa Chud is the most dangerous drug”) (for more details, see http://pca.fda.moph.go.th/public_media_detail.php?id=6&cat=40&content_id=835). In addition, there is an initiative to promote “Safe Grocery Stores” under the project “Protection on Healthcare Products and Services”. This initiative has offered training workshops for the owners of grocery stores to encourage them to only sell safe drugs and health products (for more details, see https://www.hfocus.org/content/2017/04/13780). However, despite 50 years of attempts to control and suppress Yaa Chud, it is still available and popular, usually through community-based outlets. In Thailand, antibiotics are defined as “dangerous” drugs, but they can still be legally dispensed by a licensed pharmacist without prescription. Thus, one of the problems for law enforcement is that the individual drugs in the packs can be legally sold without a prescription by a licensed pharmacist. Furthermore, the type of drugs in Yaa Chud are not easy to identify by their appearance. Thus, the consumer literally does not know what they are buying or the potential danger of a given Yaa Chud packet.

Earlier studies in low- and middle-income countries found that there are several factors related to the use of self-medication. They include the health service system, law and regulations, socio-economic factors, easy access to drugs, and mild illnesses [5, 21–26]. The poor quality of the health-care service system, waiting times and its relative high cost discourage health-care utilization. Thus self-medication is one option through which people can seek a potentially good, cheaper and more convenient service [5, 22, 27]. The use of self-medication is also related to a lack of enforcement of law and regulations, which enables easy over-the-counter access to medicine [27]. Improving socio-economic factors in LMICs lead to an increase in individuals’ decision to participate in health care and also the rate of self-medication [5, 22, 25]. The literature reveals that easy access to drugs reflects the greater availability of drugs, which allows people to have more choice to seek drugs as well as increases acceptance of self-medication [28]. The perceived severity of illness will determine the degree with which people consider the health-care option [27].

In Thailand, there have been some previous studies on Yaa Chud, but they mainly focused on its availability [10, 11, 16]. Some studies in small areas focused on factors related to the use of Yaa Chud. These factors included easy access, low price, effectiveness, health status and barriers to accessing government health services. However, those studies focused only on users’ views [29, 30] . Thus, the present study aims to identify factors associated with access to and use of Yaa Chud at the community level, based on the views and experiences of both users and suppliers. This can help to discern how and why people still seek out Yaa Chud while also raising awareness of the danger of Yaa Chud. The study concludes with a call for appropriate policy recommendations to curtail the unsafe use of Yaa Chud.

### Conceptual framework

A number of models have been used to describe and understand access to and use of health-care services, including the Behavioural Model of Healthcare Utilization. This model has mostly been used to describe and discern both individual and contextual determinants of health services utilization. In addition, the model aims to expose inequality of access and use of health-care services [31–33]. This paper takes the Behavioural Model of Healthcare Utilization as a means for understanding access to and use of Yaa Chud in Thailand.

The Behavioural Model of Healthcare Utilization was developed in the late 1960s [31] and has been revised several times [32–34]. The model initially emphasized three main factors of utilization, namely, (i) predisposing; (ii) enabling; and (iii) need factors, which are mainly related to the use of secondary care services [31]. Predisposing factors include contextual factors (e.g. health service system, laws and regulations) and an individual’s predilection to access and use a given service. These factors include socio-demographic characteristics, beliefs, and psycho-social attributes. Enabling factors deal with available means, knowledge and ability to act, including availability of services, regular source of care, financial status, etc. The “need” factors mainly focus on the evaluated or perceived health condition that requires care [33]. The use of services is generally measured in terms of use or not use, frequency of use and cost of health-care services [35, 36].

**Fig. 2:**
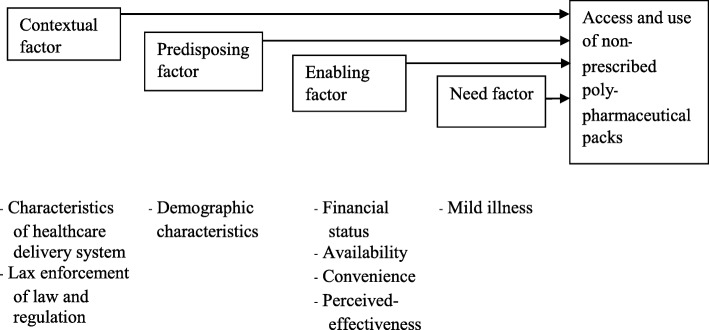
Conceptual framework for access to and use of non-prescribed poly-pharmaceutical packs in the community setting

Since access to and use of Yaa Chud are a specific manifestation of utilization as an informal health-care option, this study needs a framework, which is shown below (Fig. 2).

## Methods

### The study site

This paper reports on a sub-study of the project on “Community-level antibiotic access and use in low- and middle-income countries: Finding targets for social interventions to improve rational antimicrobial use”, known as the ABACUS (AntiBiotic ACcess and USe) study. Its aim is to compare sociocultural determinants of appropriate access and use of antibiotics among six different health and demographic surveillance sites in low- and middle-income countries in Asia (Bangladesh, Vietnam and Thailand) and Africa (Mozambique, Ghana and South Africa). For more details, see http://www.indepth-network.org/projects/abacus. This paper utilizes data from the Kanchanaburi Health and Demographic Surveillance System (HDSS) in Thailand, one of the participating ABACUS sites.

Data were collected during the period July 2016 to June 2017 in urban and semi-urban communities of the Kanchanaburi HDSS, located in western Thailand. The Kanchanaburi HDSS is one of the INDEPTH (International Network for the Demographic Evaluation of Populations and Their Health in Developing Countries) community-based study sites. The Kanchanaburi HDSS is identified as a site located in a lower/middle-income country, which was set up in 2000 with a cohort size in 2004 of approximately 43,000 people. This study, however, focuses only on urban and semi-urban areas, which consist of about 8,000 persons [37].

### Data collection techniques

Qualitative data were collected by (i) in-depth interviews (IDIs) with 18 drug suppliers and 16 community members; (ii) six focus group discussions (FGDs) with community members; and (iii) inventories of 17 drug suppliers using a standardized set of questions (see Additional file 1). Drug suppliers consisted of public and private outlets.

The IDIs, FGDs and inventories were conducted by MS, SP and WJ. These researchers have training and experience in qualitative research, and they have participated in the Kanchanaburi HDSS since 2000. The core questions used in this study were also used in all of the other INDEPTH ABACUS study sites.

### In-depth interviews (IDIs)

Antibiotic dispensers (suppliers) from both public and private health facilities and community members were the key informants for this study. This study was designed to collect data from both public and private health providers, which are at the three levels of health-care units: primary, secondary and tertiary. For the public health facilities, the samples were purposively selected. The criterion for this sampling was public health facilities that located in the Kanchanaburi HDSS area or the public health facilities that majority of the Kanchanaburi HDSS members can conveniently access. At the tertiary and secondary levels, all the public hospitals located in the Kanchanaburi HDSS were selected. The three selected samples were provincial, district and general public hospitals. The key informants were the pharmacists whose main responsibility is to dispense antibiotics. At the primary level, there were three selected sub-district health promoting hospitals, which the nurses who responsible for drug dispensing were key informants.

For the private health facilities, the samples were also purposively selected based on the same criterion set for those of the public health facilities. Although there are three selected private hospitals in this study area, only two private hospitals were willing to participate in this study. One private hospital declined to participate due to being uncomfortable with the aims of this study. For the selected private clinic, it relied on individual networks, one private clinic run by a physician, the selection was based on the existing network where one of the researchers used to use the service. Another private clinic run by a nurse was selected through networks of a key informant in a sub-district health promoting hospital.

For the private pharmacy shops, the owner or the pharmacist was the key informant. The staff of the provincial health office assisted in the selection of shops based on their full list of licensed pharmacy shops located in the Kanchanaburi HDSS. Six pharmacy shops were purposively selected and they were willing to participate in this study. Furthermore, the researchers asked for co-operation from our local networks, including village health volunteers and the village head, to help in purposively recruiting owners of grocery stores that also sell drugs. The research team clearly explained to our local networks the aims of the study. The local networks explained the project and the IDI guideline to eligible participants, and consent was received before an IDI was conducted. Two owners of grocery stores declined to participate because they felt uncomfortable talking about Yaa Chud. They were replaced by another two shops, which were recommended by village health volunteers in the communities. The interview guidelines included questions about daily experiences with sales of medicines, types of customers, antibiotic resistance and regulatory issues.

At the community level, residents were selected by simple random sampling for the IDIs. There are two sources of data that were used as a sampling frame: a list of household members in the Kanchanaburi HDSS, and a list of clients who received antibiotics from a hospital during the year before the interview. Next, the research team established a new sampling frame by matching cases of those who currently lived in the Kanchanaburi HDSS area with those who had used antibiotics in the past year. Simple random sampling (using the SPSS simple random command) was employed. The eligible persons were selected according to the designated number, age and sex of the respondents. We also randomly selected another 50% of the designated number as a reserve sample. The resulting sample includes two males and two females aged 18 to 59 years, two males and two females aged 60 years or older, and eight mothers or guardians of children aged less than 5 years.

The research team contacted the village health volunteers and community leaders by phone and in person. WJ gave the list of eligible respondents. The village health volunteers and community leaders met the eligible respondents in person and asked for their willingness to participate in the study after they had read the IDI guideline questions and consent form. The researchers then met the respondents in person and conducted IDIs.

We gathered information on experience with obtaining and using medicines by community members, focusing on where medicines are obtained, how they are used and for what reasons. These included questions related to the issues of accessing treatment, supplier/sellers of medicines, and medicines.

### Focus group discussions (FGDs)

To recruit the participants for the FGDs, the researcher used a similar method as that used for recruiting community members to participate in the IDIs. The six FGDs each included six to eight participants. The first two groups included males and females, separately, aged 18 to 29 years. The second two groups consisted of males and females, separately, aged 30 years or older. The other two groups were comprised of village health volunteers and the elderly. Participants for FGDs were recruited by the community network in the study areas. The guideline for the FGDs was also standardized and had been used in all sites of the INDEPTH ABACUS study. The core questions of the guideline consisted of issues related to accessing treatment, medicines, suppliers/sellers of medicines and antibiotic resistance.

### Inventories

Data were also collected through inventories of 17 suppliers’ outlets, including information on availability and quality of the medicines that were sold to members of the community. This inventory involved checking for the presence of all brands of five particular medicines, namely, amoxicillin, amoxicillin/clavulanic acid, chloramphenicol, ciprofloxacin and sulfamethoxazole-trimethoprim, and packaged with other drugs. Suppliers included both public and private suppliers. Public suppliers consisted of six primary, secondary and tertiary hospitals. Private suppliers included two private hospitals, one private clinic run by a physician, six pharmacy shops and two grocery stores. Since we conducted inventories and IDIs with suppliers at the same time, the key informants for inventories were the same as those for the IDIs.

### Study procedures

The researchers conducted IDIs and FGDs at places and times convenient to the participants. MS, SP and WJ conducted the IDIs and MS moderated the FGDs with assistance from SP and WJ. We used guidelines for IDIs and FGDs, with optional probes to encourage dialogue in Thai dialect. The IDIs took around 45–60 min, while FGDs lasted around 60–90 min. The inventories took 20–30 min. Each interview and FGD was audio-recorded if participants consented. Otherwise, only note-taking was done. In our study only one participant did not give permission to audio-record the IDI, but she gave alternative consent for the interview to be recorded in writing by WJ.

### Data analysis

All audio recordings were transcribed for data analysis. Line-by-line coding was performed using the Open Code programme [38]. The analysis began with searching for the Thai slang word for non-prescribed poly-pharmaceutical pack/s (i.e. “*Yaa Chud*”) in the transcriptions. Next, the researchers performed thematic analysis based on the theoretical framework. Then, codes were assigned to the material based on the themes identified in the study framework shown in Fig. 2, and, when themes emerged inductively, these were discussed and incorporated in the analysis as appropriate. The research team achieved saturation during data collection, since there was no new issue about Yaa Chud arising. Thus the thematic saturation was determined by MS and SP. In addition, the 32-item Consolidated Criteria for Reporting Qualitative Studies (COREQ) checklist was applied [39].

## Results

The results include a description of the characteristics of the participants and major themes based on the study framework, including contextual factors, predisposing factors, enabling factors, and need factors.

### Characteristics of participants

Although there were 79 individuals in the project, this study consisted only of the 49 individuals who talked about Yaa Chud and participated in the FGDs and IDIs, including both community members and suppliers. Community participants comprised 13 males and 23 females. Most had completed primary school and were aged between 20 and 79 years. Thirteen suppliers participated in this study, including eight females and five males. Most had completed a bachelor degree in pharmacy. The age of suppliers ranged from 29 to 54 years.

### Thematic analysis

Based on the conceptual framework, four themes were identified. These were contextual, predisposing, enabling and need factors. The theme concerned with context described the characteristics of a health-care delivery system that discourages the use of public health-care services alongside the lax enforcement of the law that enables access to Yaa Chud. The theme concerned with predisposing factors emphasized the demographic characteristics of those who accessed and used Yaa Chud. The third theme, on enabling factors, focused on the issues that facilitated community members to access and use Yaa Chud. The last theme described the need for Yaa Chud by community members.

### Theme one: contextual factors

This theme consists of the characteristics of the health-care service system that influence the use of YaaChud, and the lack of enforcement of laws and regulations that enables its use.

#### How do characteristics of the health-care delivery system affect access to and use of the formal health-care option?

Health-care service delivery in Thailand involves both the public and private sectors, and it includes primary, secondary and tertiary health-care units (see Additional file 2). Characteristics of the health-care services in this study are considered as predisposing factors that enable or prevent the community members from receiving health-care services. Community members reportedly often avoid the formal health-care system because of the congestion at the public facilities, long waiting times, inconvenience, and the perceived attitudes of health-care personnel. Thus, some of these people turned to Yaa Chud as a quick and more convenient alternative for some conditions.

The congestion at the public health facilities discouraged patients from asking questions of the health-care personnel. This reluctance was because they did not want to disturb the staff and they also did not want to use time that was needed for other patients. The following are quotes from elderly participants in the FGDs:

*I was afraid to ask any questions of the health personnel because it would take time while there were many patients still in the queue* (FGD community members, elderly, semi-urban*)*

Time spent while waiting to be seen by a doctor is another factor that discourages community members from using public health facilities. This is due to the congestion of the facilities. Some mentioned that they spent half or an entire day waiting to be served at some public health facilities.

*I had to wait for half a day just to get medicines when visiting the doctor for follow-up* (FGD community members, elderly, semi-urban)

The inconvenience of the health-care outlets also discourages the use of public health facilities. For example, the public outlets usually require many steps in the process of providing care. These include presenting documents, filling out forms for a new registration or loss of the hospital card, receiving a queue number, waiting in the queue, presenting all documents at the registration room, waiting for the patient chart, being screened for basic illness by a nurse, seeing the doctor, getting a prescription, paying the fee, and receiving the medicine.

*The majority of people prefer private clinics because the service is more convenient, unlike public health facilities* (FGD community members*,* village health volunteer, urban)

The perceived negative attitudes of some health-care providers created an unwelcoming environment for some community members, particularly at public health facilities. These attitudes made them feel uncomfortable when receiving service at the health facilities.

*Sometimes, they used inappropriate words* (FGD community members, females aged >18 and < 30 years old, semi-urban)

There is a clear difference for community members between receiving services from the public and from private health facilities. They reportedly felt more comfortable when receiving services from the private health sector.

*Staff of the private clinic were more welcoming* (FGD community members*,* village health volunteer, urban)

#### Does lax enforcement of laws and regulations enable Yaa Chud?

Lack of law enforcement is also another factor that influences the availability and access to Yaa Chud in the community. Although Yaa Chud is illegal, it is still available in some community outlets. Some of the participants mentioned that strong law enforcement and regular inspection would help to reduce the distribution of Yaa Chud.

*Without a strong law and enforcement to genuinely ban such medicines, Yaa Chud will never be eradicated from Thai society (*IDI supplier, female aged 40, public hospital, urban)

*There is no regular inspection; the inspector will only come to check when there is a report of selling Yaa Chud* (IDI supplier, male aged 30, grocery shop, urban)

Regarding laws and regulations, there are different points of view between formal suppliers, informal suppliers and community members. Formal suppliers argued that Yaa Chud must be eradicated, and almost all of them supported the prosecution of those who sell Yaa Chud.

*Those pharmacy shops that sell Yaa Chud need to be prosecuted* (IDI supplier, female aged 42, pharmacy shop, urban)

It is known that Yaa Chud mostly contains antibiotics and steroids. Thus, the resistance to Yaa Chud came mostly from the formal suppliers, both public and private. This is due to the fact that they know the potential danger for the consumer, and that widespread use of Yaa Chud will cause antibiotic resistance for society at large. One of the suppliers observed that some community members take Yaa Chud every day in order to relieve pain.

*There was a patient who was vomiting blood. I asked him what medicine he was taking. He said he bought it from the grocery store. I asked him how long he had been taking that medicine. He told me that he took it every day to treat pain* (IDI supplier, male aged 40, private clinic, urban)

*In my opinion, antibiotic resistance is caused by using Yaa Chud because patients get an incomplete dose of antibiotic. They may take one or two doses and stop when they feel better* (IDI supplier, female aged 42, pharmacy shop, urban)

Although safe Yaa Chud among participants was recognized as medicine that is not harmful to people, there are contradictory views of “safer” Yaa Chud among formal and informal suppliers as well as community members. The formal suppliers felt that “safe” Yaa Chud is an unrealistic goal since it is too easy to manipulate into something unsafe, which can then be sold for profit. There were strong recommendations not to use Yaa Chud in any form.

*It is impossible for Yaa Chud to be safe because we do not know what it is in the packet* (IDI supplier, female aged unknown, public hospital, urban)

The community members felt that Yaa Chud is still a necessity, though it should be safe. Those who supported the safe use of Yaa Chud included some community members and informal suppliers. They made the case that labelling Yaa Chud could promote safe use. People need to know what medicine they are taking. One grocery shop owner explained that legal and safe Yaa Chud is necessary:

*We need legal Yaa Chud that is safe to use. Labelling it as medicines that we got from the public hospital may be a safer way to use Yaa Chud* (IDI supplier, male aged 50, grocery shop, urban)

### Theme two: predisposing factors at the individual level

#### Who still accesses and uses Yaa Chud?

This theme reflects the characteristics of community members who are more likely to access and use Yaa Chud. This study found that members of the older generation and migrants who work in construction were more likely to seek and use Yaa Chud.

The older generation still uses Yaa Chud as their first-line health-care option. It may be easy and convenient for them to access the medicines because they sometimes have limited mobility. One participant mentioned that her mother accepts only Yaa Chud for treatment when she is sick.

*My mother never goes to the hospital; she only buys Yaa Chud* (FGD community member, female aged >18 and < 30 years old)

A clear generational gap was identified with respect to knowledge about Yaa Chud and its potential health risks. Although the older generation often prefers to use Yaa Chud for self-treatment, the young generation seems to avoid such medicine. One of the suppliers mentioned that the young generation may know the dangers of Yaa Chud.

*The younger generation knows it is not good and, thus, do not use Yaa Chud. Only uncles and aunts who are from the older generation still prefer such medicine* (IDI supplier, female aged 34, pharmacy shop, urban)

In addition to older people, migrant workers also have a tendency to buy Yaa Chud. Workers from the construction sector in particular seemed to need Yaa Chud for pain. They perceived this Yaa Chud as effective and available in the community outlets. The owner of one grocery shop mentioned that migrant workers frequently asked for Yaa Chud.

*Those who ask for Yaa Chud, they are not community members; they are migrant workers from a construction company* (IDI supplier, male aged 50, grocery shop, urban)

### Theme three: enabling factors at the individual level

#### How are access to and use of Yaa Chud facilitated?

This theme is concerned with factors that facilitate access to and use of Yaa Chud. This study found that a person’s economic situation, availability of Yaa Chud, convenient access to Yaa Chud, and perceived effectiveness facilitate access to, and use of, such medicines.

Lower economic status seems to be associated with a preference for Yaa Chud, since it is perceived as the most cost-effective option. It is observed that the cost of one pack of Yaa Chud is only one-fifth of the fee cost for the universal health coverage scheme (approximately 1 USD). Several participants described the price of Yaa Chud as cheap.

*It (*Yaa Chud*) is cheap; only six baht (0.2 USD) per package"* (FGD community members, elderly aged 60+, semi-urban*)*

In terms of availability of Yaa Chud, the inventories conducted as part of this study did not find Yaa Chud in stock. Generally, the pharmacy shops work under Thailand’s National Drug Act. Selling Yaa Chud is a violation and owners will face a substantial jail sentence and fine if they stock it (see Additional file 3). However, one grocery shop’s owner reported that he used to sell Yaa Chud and the retail pharmacy prepared drugs in an individual pack for him, then he made a package of Yaa Chud with multiple medicines. One supplier also mentioned that the community members could buy Yaa Chud from both grocery and pharmacy shops. This was corroborated by the community participants in the study.

*I just told the pharmacy shop how many packets of Yaa Chud I needed − for instance, 10 packets for fever. The shop would prepare 10 pills of each color in a separate bag. After that, I would pack them together (one of each color) in a small plastic bag* (IDI supplier, male aged 50, grocery shop, urban)

*They could buy Yaa Chud from grocery stores and pharmacy shops; they come in plastic packages* (IDI supplier, female aged 30, public hospital, urban)

*The seller told me that it is the best-selling package; the shop prepares those packs every two to three days and they sell out quickly* (IDI community member, female aged 65, urban, grandmother taking care of children aged <=5 years)

Since selling Yaa Chud is illegal, access to it in the community is mostly done through users’ networks. The sellers know their Yaa Chud customers and they only sell to them. If a new customer comes to buy Yaa Chud, they only sell to those accompanied by a regular customer. The owner of one grocery shop told a story of how selling Yaa Chud occurred.

*They know where to buy Yaa Chud, and the sellers’ identities are not disclosed*. *They remember who their customers for Yaa Chud are; if they do not know a customer they do not sell it* (IDI supplier, male aged 50, grocery shop, urban)

Convenience of access to Yaa Chud is a significant enabling factor that entices community members to use Yaa Chud. This includes the short time spent on obtaining it, the proximity of the supplier to their residence and freedom of choice. One of our participants reported that she only walked to the grocery shop and bought Yaa Chud that she wanted, while another participant mentioned that he just asked for Yaa Chud from the seller.

*If I want to buy Yaa Chud, I just walk to the grocery shop nearby* (IDI community member, female aged 68, semi-urban)

Community members view Yaa Chud as a viable health-care option and recognize its effectiveness. The overriding motivation is to cure the illness quickly. The participants discussed the perceived effectiveness of Yaa Chud and most mentioned the speed with which the illness can be cured.

*I got five packs and it took only two days to cure me* (FGD community members, male aged >30, semi-urban)

The formal suppliers confirmed the perception of the community members about Yaa Chud. Since the community members want to be cured quickly, then they look for the health-care option that responds to their need quickly. One of the suppliers reported that even her mother needed Yaa Chud because she wanted a speedy recovery.

*They felt that it (*Yaa Chud) *could help them get a quick cure; with repeated use, some people seemed to become addicted to these medical packs*. *Sometimes, my mom had pain from an unknown cause; so she just wanted anything that could help get rid of the pain quickly. Thus, Yaa Chud was her first choice* (IDI supplier, female aged 30, public hospital, urban)

Community members have heard about the dangers of Yaa Chud and some have experienced adverse effects. That said, some feel that the benefits of Yaa Chud outweigh its risks. Those with allergies to some of the medicines that might be in Yaa Chud feel particularly vulnerable since they do not know what medicines are in a given packet. One community member reported that her daughter experienced the dangers of Yaa Chud and she learnt about the risk of unknown medicine.

*Recently, my elder daughter ( aged 12) got a cold and my sister fed her our last Yaa Chud packet. But she had an allergic reaction to one of the drugs. It was serious because her eyes and mouth became swollen. We took her to get an anti-allergy drug. We did not know whether the drug had expired or not because there was no information on the package* (IDI community member, female aged 36, urban, mother taking care of children aged <=5 years old)

*I was allergic to something in the Yaa Chud I bought, but I did not know what medicine it was. I had swelling in the face, nausea and vomiting* (FGD community member, female aged > 18 and < 30 years old)

Notwithstanding awareness of the potential harm of using Yaa Chud, some community members seemed to accept the risk−benefit trade-off: they knew about its dangers but they still needed it in order to relieve pain and to cure their ailments.

*Actually, it is not good to take Yaa Chud. Some people know its dangers and try to limit their use. I also do not sell it because it is dangerous. Anyway, I found out that it was effective in treating pain because I took it sometimes when I had severe pain* (IDI supplier, male aged 30, grocery shop, urban)

### Theme four: need factors at the individual level

#### Why do people need Yaa Chud?

Members of the community evaluated their general health status before deciding to use Yaa Chud. Usually they opted for Yaa Chud when they perceived that they only had a mild sickness. Typical symptoms included cough, cold, fever and aches*.* One of the participants reported that her family members took Yaa Chud when they had fever or cough and she realised these are not a serious illness.

*Our family members do not like going to see a doctor, except when we have a serious sickness. If someone has a fever or cough, we buy Yaa Chud from the drugstore* (IDI community member, female aged 36, urban, mother taking care of children aged <=5 years old)

In addition, the community members felt that they could get the specific medicine they wanted for their illness. Yaa Chud seems to play a role in symptom relief among the community members. One of the participants told how she could get Yaa Chud for fever and muscle pain. *I just told the seller that I needed one packet of Yaa Chud for fever and one pack for muscle pain* (IDI community member, female aged 65, urban, grandmother taking care of children aged <= 5 years old)

## Discussion

Over the past 50 years, the Thai government has attempted to control and suppress the use of non-prescribed poly-pharmacy packs (Yaa Chud*)*, but consumer demand means that the packs continue to find their way into local communities. Thus, it is important to understand why the demand for Yaa Chud continues to be strong, particularly for certain groups of people, despite its dangers and ill-effects. This study drew partly on the Health Behaviour Model of Healthcare Utilization and confirmed that this model can be applied to both the formal and non-formal health-care settings. This study found that consumers can access inexpensive Yaa Chud from local pharmacy shops and grocery stores in their home neighbourhoods. The access and use of Yaa Chud were also associated with several factors, including predisposing factors at the contextual and individual levels, enabling factors, and need factors.

Contextual factors can affect the patterns of service use of people [32] in terms of encouraging or discouraging the use of the formal health-care sector. Our findings reveal that predisposing factors at the health facility level seemed to discourage some members of the community to seek services in the formal health-care system. Negative aspects of the public health outlets include congestion, long wait times, inconvenience and the negative attitudes of health-care personnel. Some of these findings are consistent with a prior study in Amnat Charoen Province (north-east Thailand), which found that reasons for using Yaa Chud included inconvenience and long wait times at the public health facilities [30]. Our findings are also consistent with the studies in other countries that indicated that a time absorbing and unfriendly environment at the health-care facility is one reason for self-medication [40–42]. Because of inconvenient service provision at public health facilities, people resort to self-medication, and one such self-medication in Thailand is Yaa Chud. Buying Yaa Chud at the neighbourhood outlet is perhaps the fastest and most convenient option to obtain what are, in their perception, “powerful” drugs. Although Thai residents have the right to subsidized health care at their local public health outlet, they still prefer informal health-care options for certain conditions, even though they have to pay out of their own pocket.

The second contextual factor is the lax enforcement of laws and regulations that enables Yaa Chud to remain accessible in the community − but often only on a “speakeasy” basis, since selling Yaa Chud is made through users’ networks. Although selling Yaa Chud in Thailand is illegal, the loosely regulated drug laws and non-regular inspection means that sellers of Yaa Chud are rarely caught and prosecuted. Previous studies in Thailand have described the extreme availability of Yaa Chud in the community through several channels, including vendors, mobile and non-mobile grocery stores, and small pharmacies [14, 19, 29, 30, 43]. Previous studies in Serbia and Latin American countries also pointed out the difficulty in controlling the sale of any potentially dangerous drug when there is poor law enforcement. A study in Kenya found that insufficient public health-care boosted demand for self-medication, particularly from the informal sector, leading to a weakening of existing drug legislation [44]. In addition, studies in Brazil and Indonesia have also pointed out that the legal issue was related to self-medication [45, 46]. Since unsafe Yaa Chud in Thailand is illegal and must be eradicated, programmes are needed that will reduce demand for Yaa Chud, while simultaneously providing affordable and safe alternatives. At the present time, it is probably futile to try to control Yaa Chud by focusing exclusively on retailers. In addition, the WH’s awareness campaigns and health education on Yaa Chud for community members and informal suppliers -- particularly grocery shop owners -- need to be continued.

This study found contradictory attitudes toward Yaa Chud. The possibility of safe Yaa Chud is still debated among formal suppliers as well as community members. Thus, it is necessary to find a compromise between law and community demand. Our findings revealed that “safe” Yaa Chud was something the community members want, but formal suppliers seem reluctant to accept the feasibility of this since the contents can be manipulated at the point of sale. Despite the attempt of the Thai government for five decades to eliminate Yaa Chud, it is still widely available in the community due to the strong demand from certain community members. Ideally, “safe” Yaa Chud should be a choice, and it should be designed based on the guidelines for the regulatory assessment of medicinal products for use in self-medication, as proposed by the World Health Organization [1, 2]. Labelling Yaa Chud with information regarding the contents (i.e. clear labelling), how to take the medicine, possible side effects, and possible interactions with other drugs, needs to be provided [1]. At the same time, eradication of unsafe Yaa Chud must be attempted through, for example, demand-reduction campaigns and improved services at public health facilities, backed up by strong drug law enforcement and regular spot inspections.

Andersen’s behavioural model reveals the interchangeable roles between predisposing factors and enabling factors. Predisposing factors generally influence people’s decision-making process regarding whether or not to use a service, while enabling factors affect people’s ability to access and afford health-care services [32]. This study confirmed that interaction between predisposing factors and enabling factors at the individual level encouraged the use of Yaa Chud. This is due to the fact that supply will always respond to demand in an open market [47]. Our findings indicate that members of the older generation and labour migrants seem to prefer Yaa Chud as their first-line health-care option. This is related not only to cost, but also to convenience and quick access. A previous study in Thailand also found that older persons and the poor were more likely to use drug sellers than formal health-care providers [10]. Although previous studies from several countries revealed mixed findings regarding the statistical association between age and self-medication [5], studies in Europe, Lebanon and China have found that the elderly were more likely to self-medicate [48–50]. The higher demand for self-medication among the elderly is not only to manage painful degenerative disease but also to fill gaps in an inadequate health service system [49, 50].

Immobility of the elderly creates a barrier for them to access the health service system [51]. As a result, Yaa Chud is a convenient option. However, self-medication among older persons can increase the problem of drug interactions, particularly in combination with prescribed medications for their chronic diseases [52, 53]. Thus, it is important to limit the use of Yaa Chud among Thai elderly. Home visits by health-care providers are especially important for those who have limited movement and who face difficulty accessing formal health-care services, and they can also help the elderly obtain proper medication for their demand. In addition, elderly people need to inform health-care providers about all the medicines they are using in order to evaluate the potential for drug interactions.

Being a migrant is one of the predisposing factors for Yaa Chud usage, because many migrants face a lack of health insurance coverage along with a demand for cheap medicine. For many, the informal health-care option is the most affordable choice. Previous studies have pointed out that migrants’ use health-care services less than other community members [54, 55], and this may cause or result in self-medication. One major reason for self-medication among migrants is the difficulty in accessing the formal health-care system, and that is mostly related to a lack of health insurance [41, 56–58]. In Thailand, local residents can access subsidized health care at their local public health facility under the national 30-baht scheme. The “health for all” scheme in Thailand provides universal coverage with the aim of ensuring equitable health-care access through a flat user fee of 30 baht (about 1 USD) per consultation [59]. However, a migrant who has not established residence in the locality would have to pay the full cost of treatment, even at a public health outlet. In addition, non-Thai migrants without a health insurance card cannot easily access the formal healthcare delivery system. In addition, these migrant workers want to recover quickly in order to minimize lost income, and they perceive that Yaa Chud can provide that solution. Our findings suggest that it is important to address the issue of Yaa Chud use among migrants. Thus, the campaign of the Thai FDA on Yaa Chud should focus on both Thais and migrants. Such a campaign would help to inform them about the potential harm of using Yaa Chud while also raising awareness of healthy alternatives. Furthermore, we need to explore solutions that will provide affordable health care to those working in Thailand through, for instance, insurance schemes.

Another enabling factor is the perceived effectiveness of Yaa Chud for a quick recovery. Perhaps the most important motivating factor for the purchase of Yaa Chud from our study is its perceived effectiveness. Results from studies in China, Iran and the United Arab Emirates found that perceived effectiveness of medicine from past self-medication can lead to the continued use of such medication [41, 50, 56]. Given the risks and benefits of self-medication, our analysis exposed conflicting views of Yaa Chud, and whether it should exist at all. Yaa Chud is recognized by both health-care providers and community members as a potentially dangerous product. However, users seem to balance its risks against the benefits, while the formal sector health-care providers only see the negative consequences of Yaa Chud. The significant point is that users continue to perceive Yaa Chud as beneficial, and they are likely to suggest it to friends and family members. In addition, loose regulation of Yaa Chud may lead people to think it is safe. To the community, the risks of Yaa Chud are not visible and they are largely unaware of them. These factors mean that Yaa Chud is likely to stay on the market in Thailand for the foreseeable future. There is an urgent need to put pressure on the government to consider a “safe” Yaa Chud option, and to ensure that there is more transparency in terms of its contents through clear labelling. At the same time, awareness-raising campaigns by the Thai FDA need to be continued. Campaigns should clearly communicate the risks of Yaa Chud in understandable language.

The “need” factor refers to how an individual evaluates his/her health status before deciding to seek health care [32]. Our study shows that members of the community evaluate their general health status before deciding to use Yaa Chud. Usually they opt for Yaa Chud when they perceive that they only have a mild sickness. The typical symptoms of Yaa Chud users include cough, cold, fever and aches*.* The findings of this study confirm that mild illness is often an indicator for self-medication [26, 35, 60, 61]. The non-serious illnesses of those who self-medicate include pain, fever and flu [62–65]. This finding is also congruent with a previous study, which found that the preferred health-care option in the community setting (e.g. shops and small pharmacies) is for minor health conditions [35]. When community members perceive that their health condition is not serious (e.g. cough, cold, fever and pain), the formal health-care option is not necessary. This reflects a demand for a tailored health-care option and with specific medicines to manage their own health problems. This is also a significant point of the study that urgently calls for safer Yaa Chud.

### Strengths and limitations

The strength of this study is that the researchers had the opportunity to speak directly to health-care providers and suppliers of Yaa Chud, as well as users who experienced and perceived both sides of Yaa Chud. Our respondents provided practical insights into how and why Yaa Chud is still available in the community.

However, this study also has limitations. Since selling Yaa Chud is illegal, the researchers could not interview owners of grocery shops who are currently selling Yaa Chud. Our findings are only from those who admit that they used to sell Yaa Chud and explained how packets of Yaa Chud were being prepared. This limitation suggests that further research is needed with access to key informants from the grocery shops who are currently selling Yaa Chud and are willing to participate in the study. Perhaps indigenous field worker sampling (IFWS) needs to be considered, since this would allow local community members, who have an advantage in reaching the target sample, to be trained as investigators [66].

## Conclusions

The Thai government has spent five decades trying to stop the sale and use of Yaa Chud. However, demand for Yaa Chud remains and it is still seen as an attractive option for self-medication in some communities. A compromise approach might be to explore the development of “safe” Yaa Chud by providing training to owners/dispensers of pharmacy shops/grocery stores, while also raising awareness about safe use of Yaa Chud for both providers and community members. The Yaa Chud package needs to identify the medicines it contains through clear labelling, and also to provide complete information on how to use it and potential adverse effects. Further, hazardous combinations of drugs in the package must be avoided. Appropriate and safe use of Yaa Chud as a first-line healthcare option may help reduce congestion at public healthcare outlets. However, regulatory compliance complemented by regular inspections are necessary to stop access and use of unsafe Yaa Chud.

## Additional files

Additional file 1 Research tool. (DOCX 16 kb)

Additional file 2 Healthcare delivery system in Thailand. (DOCX 12 kb)

Additional file 3 Policy to control Yaa Chud and list of antibiotics by types. (DOCX 20 kb)

## Data Availability

The research tool is described in Additional file 1. The corresponding author will provide the data set upon reasonable request.

## Abbreviations

ABACUS: AntiBiotic ACcess and Use
FDA: Thai food and drug administration
FGDs: Focus group discussions
HDSS: Health and Demographic Surveillance System
IDIs: In-depth Interviews
INDEPTH: International Network for the Demographic Evaluation of Populations and Their Health in developing countries
LMICs: Low and Middle Income Countries
NSAIDs: Non-steroidal anti-inflammatory drugs
