# Supplementary material for: What is in the drug packet?: access and use of non-prescribed poly-pharmaceutical packs (Yaa Chud) in the community in Thailand
Source: BMC Public Health. 2019 Jul 22;19:971. doi: 10.1186/s12889-019-7300-5 (PMC6647088; doi:10.1186/s12889-019-7300-5)
Supplement: Supplementary file 1 — Research tool. (DOCX 16 kb) [file 12889_2019_7300_MOESM1_ESM.docx]

**Research tool**

Material used in this paper was derived from transcripts including the following questions:

IDI Suppliers:

1. Please tell me briefly about the range of different medicines that you sell. Which are the popular ones?
2. Do you think that medicine sellers in this community ever feel encouraged to sell their customers antibiotics that may not be needed? If so, is this encouragement related to pressure from the customers or from the suppliers? Do the sellers go ahead and sell the medicines in such situations, or not? Details.
3. Do you think you have a good understanding of what antibiotic resistance is, how it is caused, and what its implications are? Is there anything on this topic that you would like to know more about? Details.
4. What do you think would be the best way to inform people (both medicine sellers and the community) about proper antibiotic use and the dangers of antibiotic resistance?
5. Overall, what do you think should be done to improve appropriate antibiotic use and decrease antibiotic resistance in this country?
6. * Do you think current government regulations are sufficient to control inappropriate antibiotic use? If not, how could things be improved?

IDIs community members:

1. What do you do when you have any of these conditions: [Probes: self-medication, health centre, pharmacy, traditional healer, grocery store etc.] and why do you choose these particular healthcare options?
2. What sort of medicines, if any, do you have at home at the moment, and what are these medicines for?
3. In general, where and how do you learn about medicines? If you wanted to know more, where would you go? What would be the best source of information about medicines for you?

FGDs community members:

1. Please describe the different alternatives that people in this community use to receive treatment if they are ill (i.e. health centre, pharmacy, traditional healer, medicine peddler, etc.). What are the main challenges people face in accessing these facilities in terms of mode of transport, time, and cost?
2. In general, how would you describe the *quality* of the health services (public and private, including pharmacies and other medicine suppliers – and referring to staff, equipment, *and* medicines) that serve your community? What, if anything, do you think could be improved?
3. How are the costs of healthcare covered by people in this community? Which are the ways of obtaining medicines, and do you have to pay for the medicines yourself? *[Probes: health insurance, charity, out-of-pocket, other?]*
4. In general, where and how do people in this community learn about medicines? What do you think would be the best way to inform people in your community about proper antibiotic use and the dangers of antibiotic resistance?
