# Supplementary material for: What is in the drug packet?: access and use of non-prescribed poly-pharmaceutical packs (Yaa Chud) in the community in Thailand
Source: BMC Public Health. 2019 Jul 22;19:971. doi: 10.1186/s12889-019-7300-5 (PMC6647088; doi:10.1186/s12889-019-7300-5)
Supplement: Supplementary file 2 — Healthcare delivery system in Thailand. (DOCX 12 kb) [file 12889_2019_7300_MOESM2_ESM.docx]

**Healthcare delivery system in Thailand**

There are three levels of healthcare delivery in Thailand, namely, primary, secondary and tertiary. The primary care unit provides basic curative care, health promotion, and prevention. This level is exemplified by sub-district health promoting hospitals. Secondary care emphasises curative care with various degrees of specialization, and is characterized by community (i.e. district) hospitals, general (i.e. provincial), and regional hospitals. Finally, tertiary care outlets provide the most specialized services and include university hospitals and large private hospitals [38-40].

The main actors in the formal healthcare delivery system in Thailand are both public and private. Public facilities cater to about three-fourths of the population. The public sector includes facilities under the Ministry of Public Health, and state organizations (e.g., universities and state enterprises). Private sector outlets include hospitals, polyclinics and clinics [38-40]. In addition to the formal healthcare delivery system, there are numerous informal healthcare providers in Thailand. These include pharmacies, drugstores, grocery stores and other alternative care providers (e.g., traditional healers). The non-prescribed poly-pharmaceutical pack, or Yaa Chud, containing medicines that are, for the consumer, of unknown identity, is available in some community-based grocery stores in Thailand.
