# Supplementary material for: What is in the drug packet?: access and use of non-prescribed poly-pharmaceutical packs (Yaa Chud) in the community in Thailand
Source: BMC Public Health. 2019 Jul 22;19:971. doi: 10.1186/s12889-019-7300-5 (PMC6647088; doi:10.1186/s12889-019-7300-5)
Supplement: Supplementary file 3 — Policy to control Yaa Chud and list of antibiotics by types. (DOCX 20 kb) [file 12889_2019_7300_MOESM3_ESM.docx]

**Policy to control antibiotics and Yaa Chud in Thailand**

Thailand’s National Drug Act has been used to control the use of antibiotics and Yaa Chud. Antibiotics have been classified by the National Drug Act as potentially dangerous drugs (yaa-antarai) since the Act was first formulated in 1967 [1]. In order to control the distribution of antibiotics, the Act indicates that legal dispensers of antibiotics are authorized health facilities, licensed physicians, pharmacists, and veterinarians. Type 1 drug stores for modern medicines (Khor Yor 1) are authorized to dispense antibiotics without prescription, and can dispense controlled antibiotics with a prescription under the supervision of a pharmacist who is physically present in the drug store. Violation of the Act is punishable by incarceration of not more than five years and a fine of not more than 10,000 baht or 312 USD [2].

Since antibiotics are defined as dangerous drugs, the national essential drug list divides antibiotics into four types which can be dispensed by different suppliers. Firstly, antibiotic Type 1 are first-line drugs that can be dispensed by all authorized health facilities. Secondly, antibiotic Type 2 includes those antibiotics that can be used when antibiotic Type 1 is not effective or if otherwise necessary. Thirdly, antibiotic Type 3 is used for certain infections by an authorized specialist. Lastly, antibiotic Type 4 is a controlled antibiotic whose use depends on several conditions such as indications for use, and prescribed by a specialist with drug utilization evaluation (DUE) [3].

Although access to antibiotics are regulated by law, they are still dispensed illegally but with unknown frequency. The National Drug Act was amended in 1987, with added measures to control the illegal distribution of antibiotics and in combination with other dangerous drugs by prohibiting the sale of Yaa Chud. Violation of this act can lead to jail time of not more than five years and a fine of not more than 50,000 baht (1,562 USD) or both. If the medicine in Yaa Chud includes a psychotropic substance, that violates the Psychotropic Substances Act, 1975. Violation of that Act imposes an additional five years of jail time and a fine of not more than 50,000 baht [2].

**List of antibiotics by type**

| Type of antibiotics | Condition of use | Sample |
| --- | --- | --- |
| antibiotic type one (ก) | the first line drug that can be dispensed by all authorized health facilities | amoxicillin, amoxicillin clavulanic acid, cloxacillin, co-trimoxazole and norfloxacin |
| antibiotic type two (ข) | will be use when antibiotics type one did not response or dispense instead of antibiotic type one if it is necessary | Lincomycin, Oflocaxin, trimethoprim, and Amikacin |
| antibiotic type three (ค) | antibiotics are used for certain infections by authorized specialist | Co-amoxiclav, Ceftriaxone, Chloramphenicol sodium succinate and Ceftazidime |
| antibiotic type four (ง) ( | a special controlled antibiotics that depending on several conditions such as indications of use, prescribed by specialist with drug utilization evaluation (DUE). | Cefixime, Levofloxacin, Vancomycin, Imipenem |

**Reference**

1. Royal Thai Government Gazette: **Royal Thai Government Gazette (ราชกิจจานุเบกษา) พระราชบัญญัติยา พ.ศ. 2510 (National Drug Act B.E. 2510 (1967)**. In*.*; 1967, 15 October (15 ตุลาคม พ.ศ. 2510).

2. Royal Thai Government Gazette: **Royal Thai Government Gazette (ราชกิจจานุเบกษา) พระราชบัญญัติยา พ.ศ. 2530 (National Drug Act (No.5) B.E. 2530 (1987)**. In*.*; 1987, 30 December (30 ธันวาคม พ.ศ. 2530).

3. Royal Thai Government Gazette: **Royal Thai Government Gazette (ราชกิจจานุเบกษา) ประกาศคณะกรรมการพัฒนาระบบยาแห่งชาติ เรื่อง บัญชียาหลักแห่งชาติ (ฉบับที่ ๒) พ.ศ. ๒๕๕๙**. In*.*, vol. 130 ( เล่ม 130); 2016, 10 November (๑๐ พฤศจิกายน ๒๕๕๙): 1-277.
